# Antithrombotic prophylaxis in a patient with nephrotic syndrome and congenital protein S deficiency

**DOI:** 10.1186/s13052-016-0227-x

**Published:** 2016-02-29

**Authors:** Andrea Artoni, Serena Maria Passamonti, Alberto Edefonti, Francesca Gianniello, Vittorio Civelli, Ida Martinelli

**Affiliations:** A.Bianchi Bonomi Hemophilia and Thrombosis Center, Fondazione IRCCS Ca’ Granda-Ospedale Maggiore Policlinico, Milan, Italy; Pediatric Nephrology and Dialysis Unit, Fondazione IRCCS Ca’ Granda-Ospedale Maggiore Policlinico, Milan, Italy; Neuroradiology Unit, Fondazione IRCCS Ca’ Granda-Ospedale Maggiore Policlinico, Milan, Italy

**Keywords:** Cerebral vein thrombosis, Pediatric, Nephrotic syndrome, Thrombophilia, Thrombolysis

## Abstract

**Background:**

Nephrotic syndrome confers an acquired prothrombotic phenotype due to the urinary loss of anticoagulant proteins.Patients with reactivation of nephrotic syndrome may develop thrombosis.

**Case presentation:**

We report the case of a life-threatening cerebral venous thrombosis in a 13 year-old boy affected by a relapse of nephrotic syndrome during a P. aeruginosa otitis/mastoiditis. Due to the worsening general conditions and the severe neurological impairment, a course of systemic thrombolysis was successfully administered, followed by anticoagulant therapy. In the present case severe inherited thrombophilia (inherited dysfunctional protein S deficiency) was identified as an important additional risk factors for thrombosis.

**Conclusions:**

A careful evalutaion of risk factos for thrombosi during reactivation of nephrotic syndrome include measurement of plasma anticaogulant proteins. When low, antithrombotic prophylaxis with heparin should be considered to prevent thrombotic episodes.

## Background

Venous thromboembolism is a rare condition in children, with an incidence of 0.7 to 1.4 events per 100,000 individuals [1] and up to 58 cases per 10,000 hospital admission [2]. Recognized risk factors for thrombosis in children are cancer, surgery, central venous catheters, thrombophilia abnormalities, local compression of the veins and systemic diseases [3, 4]. Among the latter nephrotic syndrome is characterized by coagulation impairment, due to the loss of the main circulating natural anticoagulant proteins of relatively low molecular weight, i.e., antithrombin and protein S [5]. Conflicting data are available on plasma levels of protein C, another naturally occurring anticoagulant protein, in patients with nephrotic syndrome. The hypercoagulable state is enhanced by increased plasma levels of fibrinogen and other procoagulant proteins, such as von Willebrand factor, factor VIII and factor V, that are retained by the kidney because of their high molecular weight. The prothrombotic imbalance has also been confirmed by the increase of thrombin generation and fibrin deposition [6]. Furthermore, it has been reported that in patients with nephrotic syndrome not only secondary but also primary hemostasis is impaired. Thrombocytosis is common, and an increased platelet reactivity, the presence of circulating platelets exposing the procoagulant phosphatidylserine and an increased expression of activated glycoprotein IIb-IIIa on the platelet surface have been reported [5].

A recent large case control study carried out in adults with nephrotic syndrome showed an approximately 3-fold increased risk of venous thromboembolism [7]. In children, the occurrence of venous thromboembolic events has been reported in several case series of patients with active disease. Zhang et al. [8] found a 19 % of prevalence of pulmonary embolism and/or renal vein thrombosis in 80 children with nephrotic syndrome that underwent total-body CT scan. Kerlin et al [9] reported that 9.2 % of 326 children with nephrotic syndrome developed at least one episode of thrombosis, all but one in the venous district. Cerebral vein thrombosis, a rare life-threatening thrombotic manifestation, can complicate nephrotic syndrome. In a case series of 34 children with thrombosis, 31.4 % of the events occurred in the cerebral veins [10]. Proteinuria, low plasma albumin levels (<2 g/dl), infections, anemia and the histological pattern of nephrotic syndrome (membranous nephropathy being the worse) are associated with an increased risk to develop thrombosis during the acute phase of the disease. The presence of antiphospholipid antibodies is an additional risk factors for thrombosis in children with nephrotic syndrome associated with systemic lupus erythematosus.

Although the vast majority of thrombotic events in children are in the venous district, expert opinions and guidelines differ as regards the optimal antithrombotic strategy. Some guidelines advice to use antithrombotic prophylaxis with aspirin in children with mild active nephrotic syndrome and with warfarin in severe cases (www.soc-nephrologie.org/PDF/epro/reference/SNI/PNDS-SNI-enfant.pdf). The efficacy of aspirin in primary prevention of venous thrombosis is not demonstrated. Moreover, such antithrombotic prophylaxis was used only in 21.5 % of patient included in a recent large Italian cohort study [11].

## Case Presentation

The patient was diagnosed with nephrotic syndrome at the age of 3 years, after the occurrence of generalized edema, proteinuria and transient kidney function impairment during an episode of bilateral bacterial otitis. Three years later a renal biopsy performed because of steroid-dependency, showed a mild diffuse mesangial proliferation with small areas of focal sclerosis in 14 % of the glomeruli. During childhood he had a steroid-dependent course, with relapses following common pediatric infections, and at the age of 6 years cyclosporin A was added to corticosteroids.

**Fig. 1:**
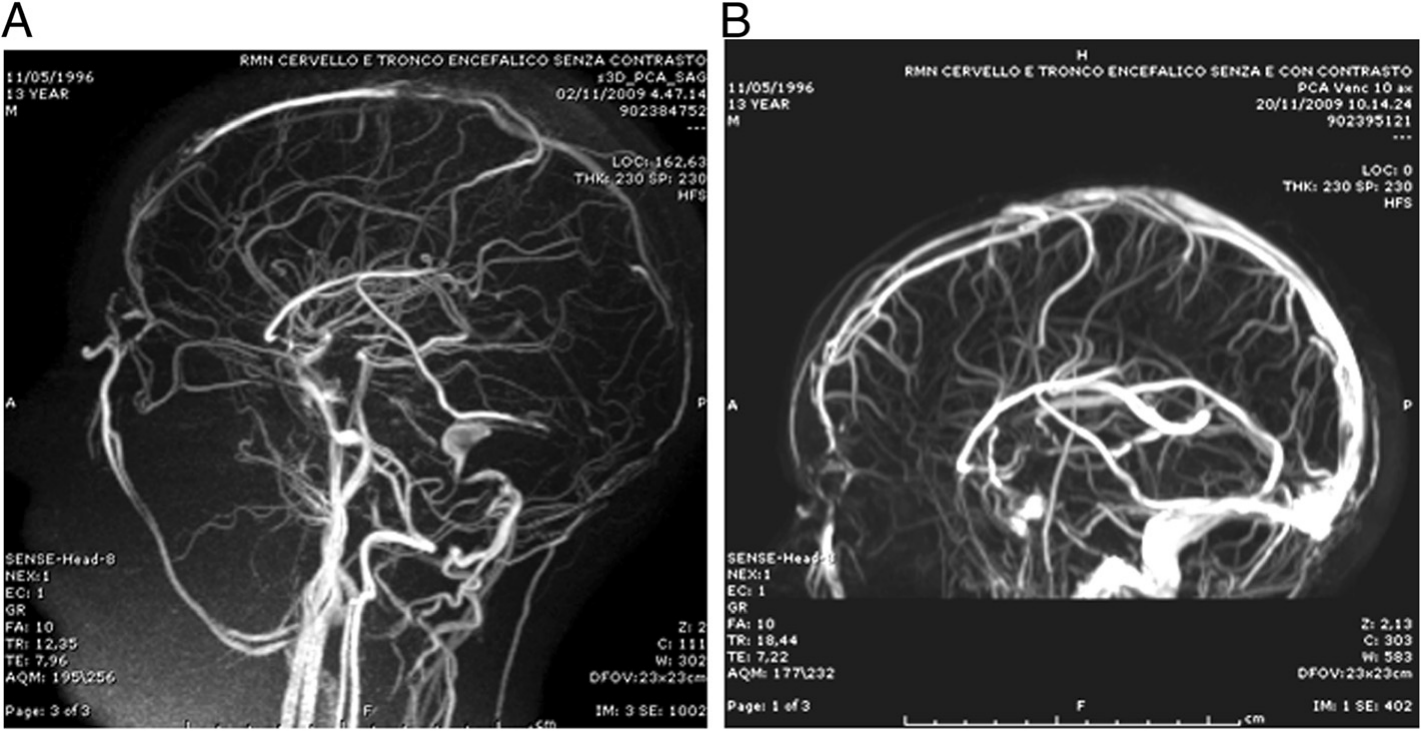
Angio-NMR before (**a**) and after (**b**) systemic thrombolysis. In panel A the superior sagittal, left trasverse and straight sinuses are not shown because occluded by thrombi, whereas in panel B is shown a complete rehabitation of the involved sinuses after therapy

No further episodes of nephrotic syndrome were reported, till the age of 13 years when the patient was admitted to the Pediatric Ward because of fever (up to 38.8 °C) and productive cough accompanied by nephrotic range proteinuria. Blood tests showed leucocytosis (15.7 WBC/mm^3^), high CRP (3.7 mg/dl), fibrinogen (558 mg/dl) and fibrin degradation products (9850 ng/ml). Large spectrum antibiotic therapy was started and the ongoing doses of prednisone and cyclosporine were increased. After few days his neurologic conditions worsened and he developed generalized seizures treated with phenobarbital. The patient was transferred to the Pediatric Intensive Care Unit as ventilatory support was needed. A cerebral angio-MR showed thrombosis of the superior sagittal, left transverse and straight sinuses (Fig. 1a), with a concomitant inflammation of the left mastoid process. Local thrombolysis through a catether in the left jugular vein was attempted, without success. Since the general conditions of the patient were deteriorating rapidly, systemic thrombolysis with recombinant tissue plasminogen activator (rtPA) was administered intravenously at the dose of 0.3 mg/kg for 6 h. A subsequent cerebral angio-MR showed a complete recanalization of the involved sinuses (Fig. 1b). In the following days intravenous heparin was given in continuous infusion (target range of activated partial thromboplastin time ratio 1.5–2.5), that was switched to low molecular weight heparin (calcium nadroparin 100 U/kg bid) when the renal function normalized. *P. aeruginosa* was isolated from the left ear swab and with a targeted antibiotic therapy his general conditions improved steadily until discharge. After few weeks phenobarbital was discontinued and the patient switched from calcium nadroparin to oral anticoagulant therapy with warfarin (target range of International Normal Ratio [INR] of the prothrombin time 2.0–3.0) for the following 6 months. The change of anticoagulant therapy from heparin to warfarin had not been done earlier because of the influence of phenobarbital on the INR.

**Fig. 2:**
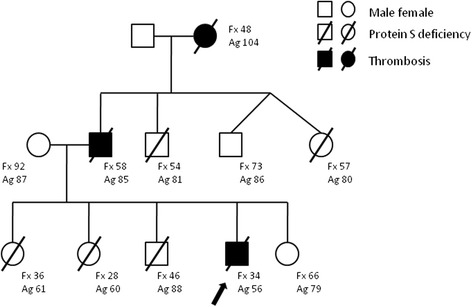
Family tree of the patient. The arrow indicates the proband. Protein S functional (Fx; normal range 61–150 %) and antigen (Ag, normal range 77–190 %) plasma levels are reported for each family member tested

Before starting warfarin the patients was tested for thrombophilia including plasma levels of antithrombin, protein C, protein S, the search of factor V Leiden and prothrombin G20210A mutations, homocysteine lupus anticoagulant and antiphospholipid antibodies (anticardiolipin and anti-β2 glycoprotein I antibodies IgG and IgM). A type II (dysfunctional) protein S deficiency was diagnosed, with normal protein S antigen and low functional levels. The inheritance of the deficiency was confirmed in first- and second degree relatives (Fig. 2). Since the episode of thrombosis the patient, who is now 17 year old, had 5 further episodes of upper respiratory tract infections, two of whom complicated by relapse of proteinuria despite immunosuppressive maintenance treatment. Antithrombotic prophylaxis with low-molecular-weight heparin (100 UI/kg od) was given in both occasions.

## Conclusions

We report the occurrence of a life-threatening cerebral vein thrombosis in a 13 year-old boy during a reactivation of nephrotic syndrome associated with bacterial otitis/mastoiditis, successfully treated with systemic thrombolysis (rtPA) followed by anticoagulant therapy. Among risk factors for thrombosis, other than inflammation and nephrotic syndrome the patient was carrier of congenital deficiency of protein S, one of the main circulating anticoagulant proteins, whose function in plasma was almost half normal. We can surmise that the low baseline plasma levels of the patients became even lower during the reactivation of nephrotic syndrome because of the urinary loss of the protein, further increasing the prothrombotic phenotype, but unfortunately we did not measure protein S at the time of thrombosis.

Antithrombotic prophylaxis with heparin is efficacious in preventing thrombotic complications. Since venous thrombosis in children is extremely rare, guidelines do not support the use of heparin prophylaxis even in high risk situations such as the first episodes or the relapse of nephrotic syndrome (www.soc-nephrologie.org/PDF/epro/reference/SNI/PNDS-SNI-enfant.pdf) [11–13] as there is no study addressing the best antithrombotic strategy. In clinical practice aspirin is often administered, although its efficacy in preventing venous thrombosis has not been clearly demonstrated so far [11]. We believe it is crucial to assess the individual profile of thrombotic risk, in order to identify patients who can benefit most from antithrombotic prophylaxis with heparin. Although congenital deficiencies of the natural anticoagulant proteins antithrombin, protein C and protein S are present altogether only in approximately 1/1000 individuals in the general population [14], one should bear in mind that these proteins can selectively decrease in plasma during the relapse of nephrotic syndrome, and therefore contribute to increase the risk of venous thrombosis. Measurement of anticoagulant proteins would help to evaluate the individual thrombotic risk and tailor antithrombotic prophylaxis. Another important patient characteristic to take into account is a family history of thrombosis. Our patient had two family members with venous thrombosis (the father and the paternal grandmother) and this strongly suggests the likelihood to detect a heritable thrombophilia abnormality. Having tested protein S at admission and started antithrombotic prophylaxis should potentially have avoided the onset of thrombosis.

In conclusion, we believe that a careful assessment of risk factors for thrombosis in children with acute nephrotic syndrome should include measurement of the anticoagulant proteins at admission and, if low, every 2–3 days until remission of the acute phase. Antithrombotic prophylaxis with heparin should be considered in those with low plasma levels of anticoagulant proteins, because of its efficacy in preventing venous thrombosis and its safety on the risk of bleeding [15].

### Consent

Written informed consent was obtained from the patient and the parents for publication of this Case report and any accompanying images. A copy of the written consent is available for review by the Editor-in-Chief of this journal.

## Abbreviations

CRP: C reactive protein
CT: computer tomography
INR: International Normal Ratio
MR: magnetic resonance
rtPA: recombinant tissue plasminogen activator
WBC: white blood cells
